# Synergistic Effect of a HER2 Targeted Thorium-227 Conjugate in Combination with Olaparib in a BRCA2 Deficient Xenograft Model

**DOI:** 10.3390/ph12040155

**Published:** 2019-10-15

**Authors:** Katrine Wickstroem, Jenny Karlsson, Christine Ellingsen, Véronique Cruciani, Alexander Kristian, Urs B. Hagemann, Roger M. Bjerke, Olav B. Ryan, Lars Linden, Dominik Mumberg, Michael Brands, Alan Cuthbertson

**Affiliations:** 1 Thorium Conjugate Research, Bayer AS, Oslo 0283, Norway; katrine.wickstroem@bayer.com (K.W.); christine.ellingsen@bayer.com (C.E.); veronique.cruciani@bayer.com (V.C.); alexander.kristian@bayer.com (A.K.); roger.bjerke@bayer.com (R.M.B.); olav.ryan@bayer.com (O.B.R.); alan.cuthbertson@bayer.com (A.C.); 2 Bayer AG, Pharmaceuticals Division, Berlin 13353, Germany; urs.hagemann@bayer.com (U.B.H.); dominik.mumberg@bayer.com (D.M.); michael.brands@bayer.com (M.B.); 3 Bayer AG, Pharmaceuticals Division, Wuppertal 42113, Germany; lars.linden@bayer.com

**Keywords:** targeted Thorium-227 conjugate, HER2-TTC, DNA damage response, Thorium-227, targeted alpha therapy

## Abstract

Targeted thorium-227 conjugates (TTCs) represent a novel class of therapeutic radiopharmaceuticals for the treatment of cancer. TTCs consist of the alpha particle emitter thorium-227 complexed to a 3,2-hydroxypyridinone chelator conjugated to a tumor-targeting monoclonal antibody. The high energy and short range of the alpha particles induce potent and selective anti-tumor activity driven by the induction of DNA damage in the target cell. Methods: The efficacy of human epidermal growth factor receptor 2 (HER2)-TTC was tested in combination in vitro and in vivo with the poly ADP ribose polymerase (PARP) inhibitor (PARPi), olaparib, in the human colorectal adenocarcinoma isogenic cell line pair DLD-1 and the knockout variant DLD-1 BRCA2 -/- Results: The in vitro combination effects were determined to be synergistic in DLD-1 BRCA2 -/- and additive in DLD-1 parental cell lines. Similarly, the in vivo efficacy of the combination was determined to be synergistic only in the DLD-1 BRCA2 -/- xenograft model, with statistically significant tumor growth inhibition at a single TTC dose of 120 kBq/kg body weight (bw) and 50 mg/kg bw olaparib (daily, i.p. for 4 weeks), demonstrating comparable tumor growth inhibition to a single TTC dose of 600 kBq/kg bw. Conclusions: This study supports the further investigation of DNA damage response inhibitors in combination with TTCs as a new strategy for the effective treatment of mutation-associated cancers.

## 1. Introduction

Targeted alpha therapy (TAT) takes advantage of the combination of the highly potent radiobiological properties of an alpha particle emitting payload and a tumor-targeting moiety such as a monoclonal antibody. The alpha particles are characterized by high linear energy transfer with a relative biological effectiveness 3- to 8-fold greater than that of X-rays [1,2]. When coupled to a suitable targeting moiety the radiation dose can be preferentially delivered to the surface of the tumor cell minimizing unwanted effects on the normal surrounding tissue.

In 2013 the first-in-class alpha emitting radiopharmaceutical Xofigo® (radium-223 dichloride) was approved by the FDA for the treatment of castration-resistant prostate cancer. The ALSYMPCA clinical trial demonstrated an overall survival benefit in patients with symptomatic bone metastases and no known visceral metastases [3]. Radium-223 is a calcium-mimetic that selectively targets hydroxyapatite in areas of high bone turnover such as bone metastases, exerting a cytotoxic effect on adjacent tumor cells through the induction of complex DNA double-strand breaks (DSBs) [4]. Due to the paucity of efficient chelators the broader application of radium-223 in antibody-based TAT is limited. In contrast, thorium-227 (half-life of 18.7 days), the parental radionuclide of radium-223, can be complexed to several chelator families including those of the 3,2-hydroxypyridinone (3,2-HOPO) class [5]. We have previously reported on several examples of targeted thorium-227 conjugates (TTCs) targeting a multitude of tumor-associated antigens including the HER2-TTC [6,7,8,9,10]. Human epidermal growth factor receptor 2 (HER2) is a transmembrane tyrosine kinase receptor that is part of the human epidermal growth factor receptor (EGFR) family [11]. The normal function of HER2 is to promote growth and proliferation, while in oncology overexpression of HER2 is a clinically validated target linked to poor prognosis and treatment outcomes in several cancer types [11].

The mechanism of cytotoxicity of alpha therapy is based on the generation of the dense ionizing track of the alpha particle, leading to a range of DNA damages including single and double strand breaks as well as clustered DNA damages [12,13]. Recently, small antibody formats have explored the use of HER2-targeted delivery of the alpha-emitter actinium-225 (half-life of 10.6 days) and astatine-211 (half-life of 7.2 h) using nanobodies or single-domain antibodies as targeting moieties [14,15]. Of those alpha-emitters available, thorium-227 is an alternative option to actinium-225 as it has a half-life of 18.7 days that matches the half-life of human IgGs in blood. Further, as thorium-227 is the progenitor of radium-223, the supply chain is established through Xofigo®. We have previously demonstrated that TTCs induce γ-H2A.X and G2/M cell cycle arrest in various cancer models indicating the involvement of DNA damage response [6,10]. Furthermore, the inhibition of multiple pathways of DNA repair has been described in the literature to have a radiosensitizing effect [16,17,18]. In this study we investigated whether the inhibition of the DNA repair proteins poly ADP ribose polymerase 1 (PARP-1) and poly ADP ribose polymerase 2 (PARP-2) might be synergistic in combination with the DNA damage inducer TTC. PARP-1 and PARP-2 are nuclear enzymes that have an integral role in base excision repair, including the detection of single strand DNA breaks and recruiting repair proteins to the site of the damage [19]. PARP-1/2 deficiency leads to the accumulation of DNA damage and a decrease in cell viability [20]. The studies presented included combination therapy with the PARP-1/2 inhibitor olaparib, which is FDA approved for treatment of BRCA-mutated ovarian and breast cancer. BRCA1 and BRCA2 are tumor suppressor genes with protein products that are central in DNA double strand repair. Defects in BRCA therefore make the cell highly sensitive to PARP-1/2 inhibition and olaparib works efficiently in patients characterized with germline BRCA mutations on the basis of synthetic lethality [21].

We report herein the preclinical data from the combination treatment with HER2-TTC and olaparib. As such, the human colorectal cancer isogenic cell line pair DLD-1 parental and DLD-1 BRCA2 -/-, the latter harboring a defect in the DNA double strand repair gene, was used. Both in vitro and in vivo experiments revealed a significant synergistic effect in the BRCA2 deficient model. This study supports the future development of new therapeutic strategies combining DNA damage response inhibitors with the DNA damage-inducing TTCs.

## 2. Results

### 2.1. Preparation and Characterization of HER2-TTC

**Figure 1:**
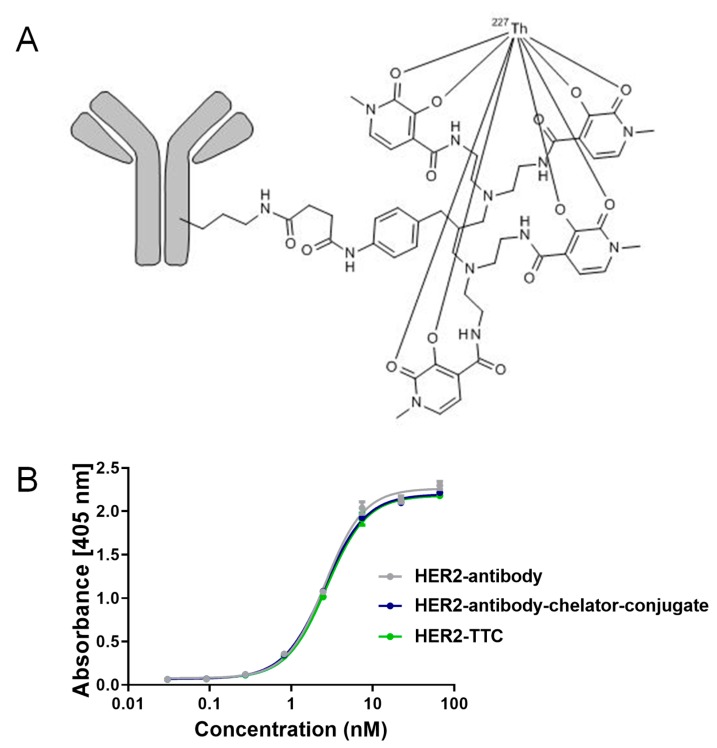
Schematic illustration of human epidermal growth factor receptor 2-targeted thorium-227 conjugates (HER2)-TTC and determination of binding affinity by enzyme linked immunosorbent assay (ELISA) assay. (**A**) Schematic illustration of HER2-TTC. An N-hydroxysuccinimide-activated 3,2-HOPO chelator was coupled to the ε-amino groups of the lysine residues of the HER2-Ab (trastuzumab), and the conjugate was radiolabeled with thorium-227 and (**B**) ELISA on recombinant human HER2. Radiolabeled HER2-TTC (2 kBq/µg) was compared to the antibody and the antibody-chelator conjugate demonstrating no change in binding affinity.

The 3,2-HOPO chelator was conjugated to free amino side-chain of lysine residues on trastuzumab via amide bond formation, yielding the HER2-antibody-chelator conjugate enabling complexation of thorium-227 [5,7,22] (see schematic illustration Figure 1A). The radiochemical purity (RCP) and radiochemical yield was determined by instant thin-layer chromatography (iTLC) and high-purity germanium detector (HPGe) for each experiment and was shown to be consistently ≥ 95%. The binding affinity of the HER2-TTC was compared against the antibody-chelator conjugate and the antibody by enzyme linked immunosorbent assay (ELISA), demonstrating that the binding affinity was not impaired by the conjugation or the radiolabeling reaction (Figure 1B). Further, in vitro stability studies were conducted which demonstrated that the HER2-TTC retains its binding affinity over the course of 48 h upon storage as indicated by the determined immune-reactive fraction of 77%. In parallel, RCP was determined to be in the range of ≥ 95% as demonstrated by iTLC and radio-HPLC experiments (see supplementary Figure S1 and Table S1). In summary, these data demonstrate in vitro stability of the HER2-TTC, enabling in vivo dose applications.

### 2.2. Synergistic Effect of HER2-TTC and PARPi Olaparib on DLD-1 BRCA2 -/- In Vitro

**Figure 2:**
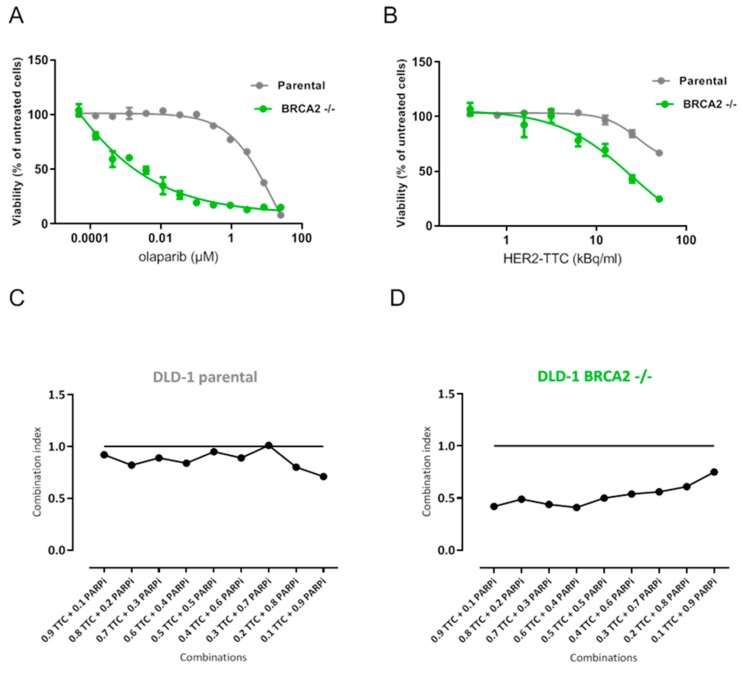
In vitro characterization of HER2-TTC and olaparib in DLD-1 parental and DLD-1 BRCA2-/- cell lines. (**A**) Single treatment poly ADP ribose polymerase inhibitor (PARPi), olaparib, on DLD-1 parental and DLD-1 BRCA2-/- and (**B**) single treatment HER2-TTC on DLD-1 parental and DLD-1 BRCA2 -/-. combination index plot for evaluation of combination with HER2-TTC and olaparib on (**C**) DLD-1 parental and (**D**) DLD-1 BRCA2-/-. The CI was determined according to the median-effect model of Chou-Talalay, with CI < 0.8 defined as synergistic effect, 0.8 < CI > 1.2 defined as additive effect, and CI > 1.2 defined as antagonistic effect [24].

The in vitro combination effect of HER2-TTC and PARPi olaparib was evaluated on the colorectal adenocarcinoma cell line DLD-1 parental and DLD-1 BRCA2 -/- Firstly, we determined the expression of HER2 antigens on the surface of the cell lines by flow cytometry. Both cell lines were found to be HER2-positive, however, in comparison to HER2-positive breast cancer cell lines, typically harboring receptor densities in the range of 50,000 to 1,000,000 receptors/cell [23], receptor densities were determined to be in the range of ~ 5000 mAb bound per cell (see Table 1), and therefore judged as low-expressing cell lines. Secondly, we evaluated the in vitro potency of the single agent, either olaparib or HER2-TTC. As anticipated, the DLD-1 BRCA2 -/- cell line was significantly more sensitive to olaparib as compared to the DLD-1 parental cell line, with a 170-fold difference in IC_50_-value (Figure 2A). DLD-1 BRCA2 -/- was also more sensitive to HER2-TTC demonstrating a 9-fold difference in IC_50_-value (Figure 2B). 

The combination effect of HER2-TTC and olaparib was then evaluated by generating IC_50_-isobolograms and calculating the combination index (CI) to determine synergy and additive or antagonistic effects [24]. HER2-TTC and olaparib demonstrated significant synergistic effect over a range of concentration ratios in the DLD-1 BRCA2 -/- cell line with an average CI value of 0.6 (Figure 2D). In contrast, the DLD-1 parental cell line gave only an additive effect with the average CI value of 0.9 (Figure 2C). 

### 2.3. Specific Tumor Accumulation of HER2-TTC in the HER2 low DLD-1 Xenograft Models

**Figure 3:**
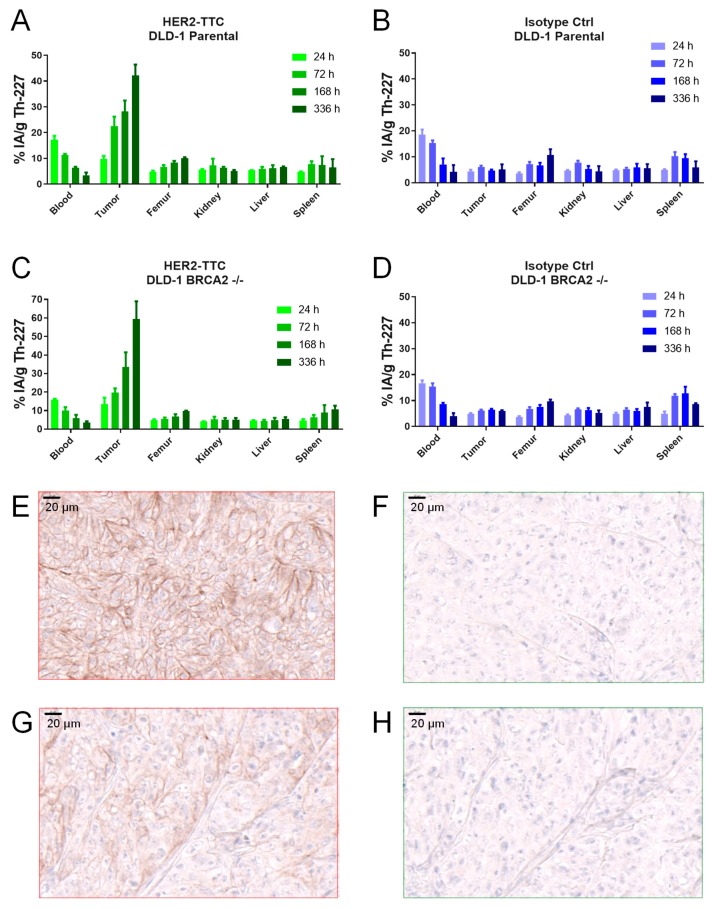
Biodistribution of HER2-TTC and a radiolabeled isotype control in mice with DLD-1 parental and DLD-1 BRCA2-/- xenograft model. (**A**) and (**C**) HER2-TTC and (**B**) and (**D**) radiolabeled isotype control 24, 72, 168, and 336 h after single intravenous dose administration (600 kBq/kg bw, 0.14 mg/kg bw, i.v.). For each time point organs from three individual animals were harvested. Thorium-227 activities were determined using a high purity germanium detector (HPGe) and expressed as percentage of the injected thorium-227 dose per gram. (**E**) HER2 IHC in DLD-1 parental tumors and (**F**) staining with respective isotype control antibody. (**G**) HER2 IHC in DLD-1 BRCA2 -/- tumors and (**H**) staining with respective isotype control antibody.

The biodistribution of the HER2-TTC was compared to a radiolabeled isotype control in the subcutaneous DLD-1 parental and the BRCA2 deficient models. Mice were administered i.v. with a single dose of HER2-TTC or isotype control of 600 kBq/kg bw at a protein dose of 0.14 mg/kg bw. Tumor accumulation of thorium-227 was observed out to 336 h, with a measured uptake of 42 ± 4% and 59 ± 10% injected activity per gram (% IA/g) tumor for the DLD-1 parental (Figure 3A) and DLD-1 BRCA2 -/- respectively (Figure 3C). Furthermore, there was no significant difference in tumor uptake when comparing the two models (Figure S2). The specificity of tumor targeting was evidenced by the low level of tumor uptake of the isotype control which reached a maximum of approximately 5% IA/g tumor in both tumor models (Figure 3B,D). Tumor accumulation of the HER2-TTC over time was accompanied by a decrease of thorium-227 in blood, with a tumor to blood ratio at 336 h of 17.3 ± 3.5 for the DLD-1 parental and 12.8 ± 2.4 for the DLD-1 BRCA2 -/- (Table 1). All calculated % of injected activity per gram are summarized in the supplementary Table S2. The IHC analysis demonstrated comparable levels of expression with a score of 1—2+ in both xenograft models. Therefore, the HER2 expression level was judged as low to medium (Figure 3E–H,G; Table 1). In summary, the biodistribution study demonstrated comparable and a significant and specific accumulation of HER2-TTC in both tumor models.

As discussed by Kozempel et al. [25], the recoil effect of alpha-particles can impact the energy deposition of the respective radiolabeled targeted thorium-227 conjugate. Since thorium-227 decays to radium-223, the analysis of the activity of radium-223 in tumors was included (see supplementary Figure S3). It was observed that more radium-223 activity was detected in tumors treated with HER2-TTC in comparison to the radiolabeled isotype control, manifesting the specificity of HER2-TTC, although the determined activity of radium-223 was lower than theoretical calculated if all activity from decaying thorium-227 was maintained in the tumor. The complete analysis of the distribution of thorium-227 and radium-223 will be addressed in a good laboratory practice (GLP) distribution study in a relevant species.

### 2.4. Significant Dose-Dependent Tumor Growth Inhibition of HER2-TTC and Olaparib in HER2 Low Expressing DLD-1 Xenograft Models after Single Dose Administration

**Figure 4:**
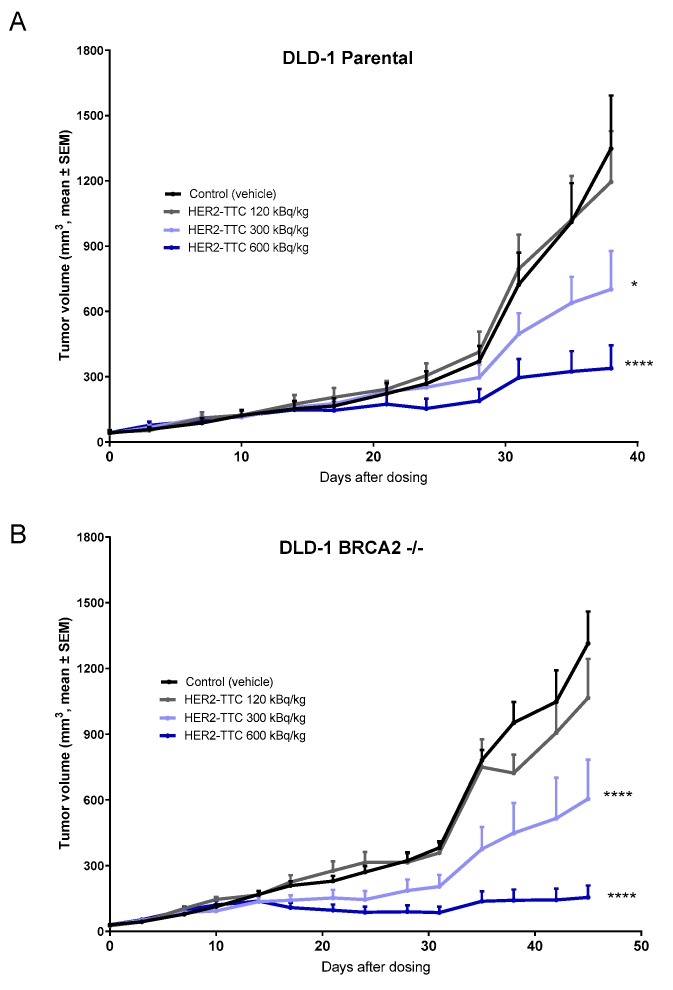
In vivo efficacy of single treatment with HER2-TTC in DLD-1 parental xenograft and DLD-1 BRCA2 -/- xenograft. (**A**) Tumor size determined after a single dose administration of HER2-TTC (120, 300, or 600 kBq/kg bw, 0.14 mg/kg bw, i.v.) as compared to vehicle in DLD-1 parental and (**B**) tumor size determined after a single dose administration of HER2-TTC (120, 300, or 600 kBq/kg bw, 0.14 mg/kg bw, i.v.) as compared to vehicle in DLD-1 BRCA2 -/-.

The in vivo efficacy of HER2-TTC in subcutaneous DLD-1 parental and BRCA2 -/- models was determined after administration of HER2-TTC at a single dose of 120, 300, or 600 kBq/kg bw in comparison to a vehicle control group. Calculations of the average doubling time for the vehicle control indicated that there was no difference in growth between the two models (Table 1). The treatment with the HER2-TTC resulted in a dose-dependent and significant tumor growth inhibition in both xenograft models (Figure 4A,B; significance presented in Table 1) compared to vehicle-treated group. Furthermore, calculations of treatment-over-control ratios indicated that DLD-1 BRCA2 -/- was more sensitive to the highest dose of HER2-TTC (600 kBq/kg bw) compared to the DLD-1 parental (Table 1).

### 2.5. Synergistic Effect of HER2-TTC in Combination with PARPi Olaparib in the DLD-1 BRCA2 -/- Xenograft Model

Based on the findings from the monotherapy studies we then explored the in vivo efficacy of the combination therapy. HER2-TTC was administered at a single dose of either 125 kBq/kg bw or 300 kBq/kg bw, the former selected as an inactive dose and the latter demonstrating statistically significant anti-tumor efficacy compared to control in both models. Olaparib dosing was performed daily at 25 or 50 mg/kg bw i.p. for 4 weeks.

**Figure 5:**
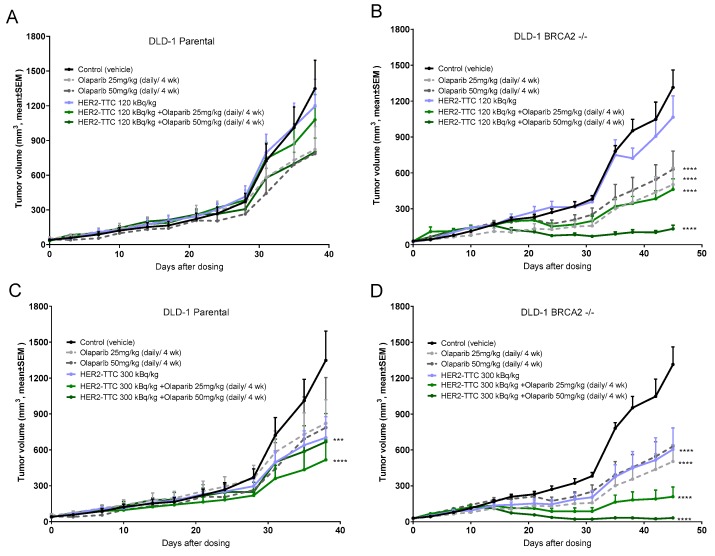
In vivo efficacy of combination treatment with HER2-TTC and olaparib in DLD-1 parental xenograft and DLD-1 BRCA2 -/- xenograft. (**A**) Tumor size determined in DLD-1 parental after a single dose administration of HER2-TTC (120 kBq/kg bw, 0.14 mg/kg bw, i.v.) and olaparib (25 or 50 mg/kg bw daily for four weeks) as monotherapies or in combination treatment as compared to vehicle, (**B**) tumor size determined in DLD-1 BRCA2 -/-, after a single dose administration of HER2-TTC (120 kBq/kg bw, 0.14 mg/kg bw, i.v.) and olaparib (25 or 50 mg/kg bw daily for four weeks) as monotherapies or in combination treatment as compared to vehicle, (**C**) tumor size determined in DLD-1 parental after a single dose administration of HER2-TTC (300 kBq/kg bw, 0.14 mg/kg bw, i.v.) and olaparib (25 or 50 mg/kg bw daily for four weeks) as monotherapies or in combination treatment as compared to vehicle, and (**D**) tumor size determined in DLD-1 BRCA2 -/-, determined after a single dose administration of HER2-TTC (300 kBq/kg bw, 0.14 mg/kg bw, i.v.) and olaparib (25 or 50 mg/kg bw daily for four weeks) as monotherapies or in combination treatment as compared to vehicle.

The effect of olaparib as monotherapy (25 or 50 mg/kg bw daily, 4 weeks) gave significant efficacy only in the BRCA2 -/- model (Figure 5). Significant anti-tumor efficacy was observed for the combination therapy in the DLD-1 BRCA2 -/- xenografts (Figure 5B,D) with tumor regressions noted across both treatment groups. Based on calculations using the Bliss additivity model, we determined that the combination effect was synergistic when using 125 kBq/kg bw HER2-TTC + 50 mg/kg bw olaparib and 300 kBq/kg bw HER2-TTC + 50 mg/kg bw olaparib [26] (Table S3). In contrast no synergy was observed in the parental model for any of the combination therapy doses evaluated (Figure 5A,C; Table S4). Furthermore, all combination treatments were well tolerated as evidenced by no significant loss in the animal body weight compared to vehicle control groups (Figure S4).

### 2.6. Effect on Hematology of the Combination Compared to Monotherapy

**Figure 6:**
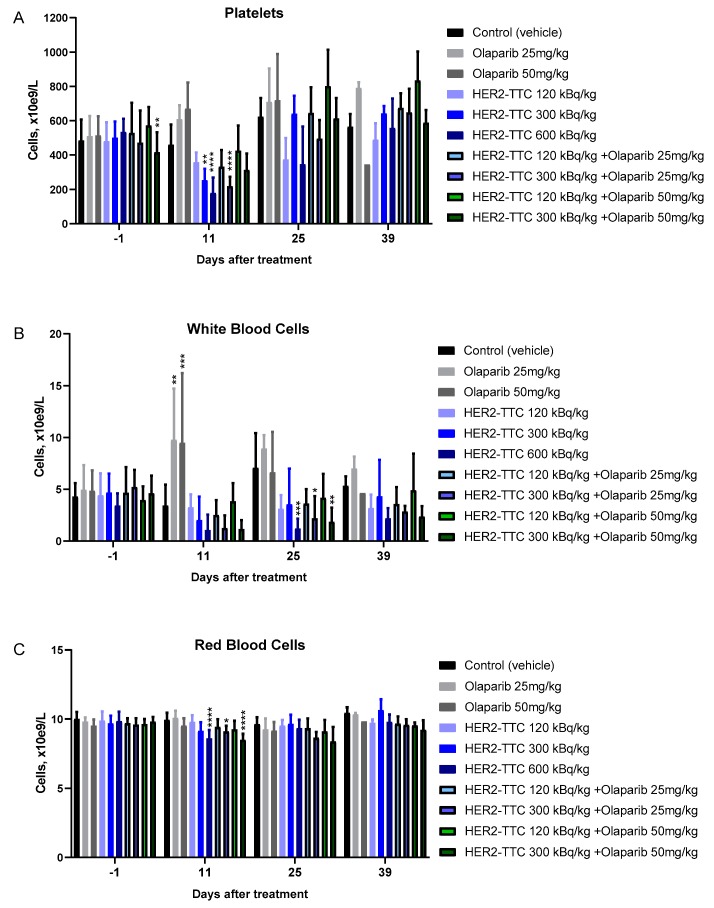
Hematology analysis in DLD-1 parental bearing mice. (**A**) Platelets, (**B**) white blood cells, and (**C**) red blood cells collected before dosing with HER2-TTC, olaparib, or combination therapy, and thereafter every other week. Analysis performed by using VetScanHM5.

Blood samples were collected prior to treatment and thereafter every other week until the study end point. The red blood cells were largely unaffected for all dosing groups (Figure 6C), while platelet counts decreased to around half the initial value for the highest dose of 600 kBq/kg bw HER2-TTC with nadir at 12 days followed by a recovery to normal values by day 26 (Figure 6A). In addition, this high dose had a marked effect on the white blood cells with nadir at 11 days followed by full recovery at day 39 (Figure 6B). There was no effect on platelets or red blood cells of the olaparib monotherapy doses. While demonstrating an equivalent antitumor activity, the combination of 120 kBq/kg bw and olaparib (50 mg/kg bw) resulted in reduced myelosuppression compared to a single dose 600 kBq/kg bw HER2-TTC (Figure 5B).

## 3. Discussion

TTCs represent a new class of systemic radiotherapeutic agents with the capability of targeting multiple cancer types. We report herein a HER2-TTC comprising three key components, the anti-HER2 monoclonal antibody trastuzumab (HER2-Ab) [27], the 3,2-HOPO chelator conjugated via amide bond formation to the free amino groups of lysine residues on HER2-Ab, and thorium-227 which forms a highly stable complex with the chelator [5,7]. Simple mixing of the conjugate with thorium-227 at ambient temperature induced high radiochemical purity (RCP) of ≥ 95% for the complex as measured by iTLC. The HER2-TTC was further evaluated by ELISA, demonstrating that the binding affinity was not impaired by the conjugation or the radiolabeling.

In the present study we evaluated the HER2-TTC together with the PARPi olaparib. PARP-1/2 is essential for maintaining genome integrity and the inhibition has been demonstrated to sensitize cells to ionizing radiation and other types of DNA damaging agents [16]. PARP inhibition leads to the conversion of single strand DNA breaks to double strand breaks (DSBs) [28]. Normally DSBs can be repaired by two pathways, HR (homologous recombination) which utilises a DNA template on the sister chromatid and is high fidelity repair or NHEJ (non-homologous end-joining) utilising non-template repair leading to errors and genetic instability [29]. In BRCA-deficient tumors, homologous recombination is not functional, and the cell is directed towards error-prone repair and cell death. As such, PARP inhibitors induce the loss of PARP-1/2 function leading to the accumulation of DNA lesions and inadequate repair of double-strand DNA breaks [30]. As the primary mode of action of the TTC is induction of complex DNA damage we hypothesized that the combination with PARPi would be synergistic.

Since olaparib is known to induce synthetic lethality in BRCA-mutated tumors [21], we selected the BRCA2-deficient colorectal cancer cell line (DLD-1 BRCA2 -/-) for this study. Identical data sets were also generated in the BRCA2-proficient parental cell line (DLD-1 parental) for comparison. First, both cell lines were determined to express a low level of approximately 5000 HER2 receptors/cell by flow cytometry. Further, a clear difference was observed in vitro with the BRCA2 -/- cell line demonstrating increased sensitivity to HER2-TTC and clear synergistic effect from the combination based on the calculated combination index compared to the parental cell line. As isogenic cell line pairs, harboring DNA repair deficiencies, are barely described and available, it has to be noted that the cell line used in the presented study served only as a tool to study combinations of HER2-TTC and olaparib.

To evaluate if the in vitro result translated to the in vivo setting we continued with the mono- and combination treatment with HER2-TTC and olaparib. Firstly, biodistribution and retention of HER2-TTC was shown to be specific as evidenced by the high tumor uptake in both models (40–60% IA/g) compared to ca. 5% IA/g for the radiolabeled isotype control at 336 h, the latter due to antibody delivery from the enhanced permeability and retention effect [31]. Further, it was observed that an amount of radium-223 is retained in the tumor after targeting and internalization of the HER2-TTC; in contrast, the radiolabeled isotype control showed less retention of radium-223 in the tumor. Interestingly significant tumor growth inhibition was achieved for the TTC monotherapy at the higher doses of 300 and 600 kBq/kg, despite the low HER2 expression levels as measured by both flow cytometry and IHC. These data may therefore support the further investigation of HER2-TTC as a monotherapy in patients with an HER2 expression level of 1+, which are not deemed suitable for either Herceptin®- or Kadcyla®-based therapies.

A strong synergistic effect was observed using the combination of the non-effective monotherapy dose of 120 kBq/kg bw in combination with 50 mg/kg bw (daily, 4 weeks) olaparib and 300 kBq/kg bw in combination with 50 mg/kg bw (daily, 4 weeks) olaparib. Both combinations resulted in a robust tumor growth inhibition. The lack of synergy observed in the parental model further reflects the specificity of the combination therapy to the BRCA2-deficient model, introducing a second layer of tumor targeting in addition to HER2 targeting. Furthermore, no significant myelosuppression was observed for the combination on the red and white blood cells as well as platelet populations. As systemic radioimmunotherapies suffer from dose-limiting toxicity to the bone marrow this study offers hope for identifying effective combinations with much reduced overall hematological toxicity. In conclusion, the data clearly indicate the potential for increasing the therapeutic window by using combination therapy with HER2-TTC and olaparib.

BRCA germline mutations account for around 5–10% of breast cancers and 10–18% of ovarian cancers [32]. Although HER2-overexpressing breast cancers appear seldom to carry BRCA mutations, ovarian cancers have significantly higher numbers of BRCA mutant/HER2 positive cases and this patient population would therefore represent a potential clinical indication for further exploration for this combination [33]. New tumor-specific antigens expressed by BRCA-deficient breast cancers may form the basis for the development of new TTCs in the future. In addition the findings reported in this paper warrant the further investigation of targeted alpha therapy approaches in combination with other DNA damage response inhibitors.

## 4. Materials and Methods

Cells: DLD-1 parental and DLD-1 BRCA2 -/- cell lines were obtained from Horizon Discovery group. The cell lines were authenticated using PCR fingerprinting by the provider. Cells were maintained in an incubator at 37 °C and 5% CO_2_. The cells were cultured in RPMI 1640 supplemented with 10% fetal bovine serum, 2 mmol/L glutamine, 25 mmol/L sodium bicarbonate, 1% penicillin, and 1% streptomycin.

Binding and receptor density: DLD-1 parental and DLD-1 BRCA2 -/- (100 000 cells, 100 µL) were seeded in 96 well plates and incubated with a titration of anti-HER2 antibody (0.0006-100 µg/mL) for one hour at 4 °C, followed by incubation with 100 µL anti-human IgG-PE (Cat# 409304, biolegend) for one hour at 4 °C. The fluorescence intensity was acquired from a Guava EasyCyte 8HT flow cytometer and the raw data was processed using FlowJo to obtain the mean fluorescence intensity values. The data was subsequently plotted using GraphPad Prim software version 7.0 against the concentration of anti-HER2-antibody. The mAbs/cell was determined making a standard curve using beads from Quantibrite (Cat# 340495, BD biosciences).

Preparations of the HER2 antibody-chelator conjugate: The synthesis of the 3,2-HOPO chelator was performed as previously described [5,22]. The 3,2-HOPO chelator was conjugated to lysine residues within the HER2-antibody and an isotype control antibody through in situ activation of the chelator using EDC/NHS chemistry and subsequent incubation with the antibody in phosphate buffered saline (PBS), pH 7.0, for 60 min at room temperature. The molar ratio used during the reaction was 1/8/8/16 (mAb/chelator/NHS/EDC). The antibody-chelator conjugate was purified by size exclusion chromatography (HiLoad 16/600 Superdex 200; GE Healthcare, Chicago, USA). A chelator to antibody ratio (CAR) of 0.8 was determined by HPLC. Conjugates were subsequently stored at −20 °C.

Radiolabeling and characterization of HER2-TTC: Thorium-227 was purified from an actinium-227 generator as previously described [34]. For in vitro studies, the antibody-chelator conjugates were mixed with thorium-227 activities ranging from 0.5–2.5 MBq and incubated at room temperature for 60 min. For in vivo studies, specific activities of 4.2 kBq/µg (600 kBq/kg), 2.1 kBq/µg (300 kBq/kg) and 0.84 kBq/µg (120 kBq/kg) were prepared using an adjusted body weight per animal of 0.03 g and a total antibody dose of 0.14 mg/kg. Radiochemical purity (RCP), defined as the amount of thorium-227 bound to the HER2-TTC, was determined by instant thin-layer chromatography (iTLC) and radio-HPLC.

Enzyme linked immunosorbent assay (ELISA): Recombinant human HER2 (Cat# 1129-ER, R&D Systems) was coated to 96-well plates (1 µg/mL; NUNC/Maxisorp). Wells were blocked with 3% BSA in PBS. Cold HER2-antibody conjugate, an isotype control antibody and the radiolabeled HER2-TTC (2 MBq/mg, stored for 72 h) were titrated (1:5; 10 µg/mL) on the HER2-coated ELISA plate. Unbound samples were washed off and bound samples were visualized using horseradish peroxidase-labeled goat anti-human lambda antibody (Cat# 2060-05, Southern Biotech) followed by visualization with the peroxidase substrate ABTS (Cat# 002024, Life Technologies, Carlsbad, USA). The absorbance was measured at 405 nm in a plate reader (Perkin Elmer, Waltham, USA).

IC_50_ isobolograms: Combination experiments were conducted with DLD-1 parental and DLD-1 BRCA2 -/-. The cells were seeded in 384 well plates (30 µL per well, 30,000 cells/mL). After 24 h the cells were treated with a titration of HER2-TTC (0.01–50 kBq/mL) and a titration of olaparib (0.01–25 µM) as single treatments and in nine different fixed-ratio combinations of HER2-TTC (C1) and olaparib (C2); 0.9 × C1 + 0.1 × C, 0.8 × C1 + 0.2 × C2, 0.7 × C1 + 0.3 × C2, 0.6 × C1 + 0.4 × C2, 0.5 × C1 + 0.5 × C2, 0.4 × C1 + 0.6 × C2, 0.3 × C1 + 0.7 × C2, 0.2 × C1 + 0.8 × C2, and 0.1 × C1 + 0.9 × C2. After five days incubation, cell viability was determined by using CellTiter Glo (CTG) 2.0 Luminescent Cell Viability Assay (Cat# G9242, Promega), according to manufacturer’s protocol. The cell viability was expressed in % by normalization to cells seeded in culture medium supplemented with buffer solution and the absolute IC_50_ values were determined by using GraphPad Prims software version 7.0, making a non-linear regression curve fit, selecting log (inhibitor) vs. response–variable slope (four parameters). The IC_50_ isobolograms were generated by plotting the actual IC_50_ values of HER2-TTC and olaparib along the x- and y-axis. The combination index (CI) was determined according to the median-effect model of Chou-Talalay [24], with CI < 0.8 defined as synergistic effect, 0.8 < CI > 1.2 defined as additive effect, and CI > 1.2 defined as antagonistic effect.

Animal models: The animal studies were managed at the Laboratory of Pharmatest Services Ltd., Itäinen Pitkäkatu 4C, 20,520 Turku, Finland with the approval of the National Committee for animal experiments (license number ESAVI-2331-04.10.07–2017). The animal experiments were conducted following Directive 2010/63/EU, the protection of animals used for scientific purposes. A 3R (Replacement, Reduction, and Refinement) principle was applied. In all studies, female NMRI nude mice (RjOrl:NMRI-*Foxn1^nu^*, Janvier Labs, France, 5–6 weeks) were used. All animals received an intraperitoneal (i.p.) injection of an unrelated murine IgG2a antibody (200 µg/animal; UPC10; Sigma) 24 h prior treatment to block unspecific spleen uptake [35].

In vivo biodistribution: One million DLD-1 parental cells suspended in 0.05 mL PBS (millipore, Cat# L1825, Burlington, USA) or five million DLD-1 BRCA2 -/- cells suspended in 0.05 mL 50% matrigel (BD Biosciences, San Jose, USA) were inoculated subcutaneously into the flank. When tumor size averaged 200mm^3^, tumor bearing mice were administered with a single intravenous (i.v.) injection of HER2-TTC or isotype control (600 kBq/kg body weight (bw), 0.14 mg/kg bw) and sacrificed after 24, 72, 168, and 336 h. Tumors and organs were harvested from three mice per group at each time point. Radioactivity was counted using a high-purity germanium detector (HPGe) linked to an autosampler (GEM-F8250, Ortec Gamma Data). To identify thorium-227 and radium-223, the GammaVision software and Npp32 analysis engine (Reg. Guide 4.16 detection limit method) were used. For thorium-227 measurement, the 235.96 keV (abundance 12.90%), 256.23 keV (abundance 7.00%), 329.85 keV (2.90% abundance), 286.09 keV (abundance 1.74%), 304.50 keV (abundance 1.15%), 334.37 keV (abundance 1.14%), and 299.98 keV (abundance 2.21%) gamma peaks were used. Thorium-227 counts were corrected to the time of injection and expressed as percentage of injected dose of thorium-227 per gram (% IA/g).

In vivo efficacy: One million DLD-1 parental cells or five million DLD-1 BRCA2 -/- cells were suspended in 0.05 mL PBS and inoculated subcutaneously into the flank. When tumor size averaged 90 mm^3^, mice were randomized into groups with 10 mice per group. Mice received a single i.v. injection of HER2-TTC (125, 300, or 600 kBq/kg bw, 0.14 mg/kg bw), radiolabeled isotype-control (300 kBq/kg bw, 0.14 mg/kg bw), HER2 antibody-chelator conjugate (0.14 mg/kg bw), or vehicle. In a parallel arm of the study, four groups were treated with HER2-TTC (125 or 300 kBq/kg bw, 0.14 mg/kg bw) in combination with olaparib (25 or 50 mg/kg bw daily, i.p. for 4 weeks)**.** Monotherapy of olaparib was included in control groups. In both arms of the study, body weights and tumor dimensions were measured twice a week during the course of the study. Blood samples was collected from the saphenous vein to 100 µL EDTA tubes before dosing (day -1 for DLD-1 parental and day -2 for DLD-1 BRCA2-/-) and thereafter every other week. Hematology samples were analyzed using VetScanHM5 (Abaxis Europe GmbH, Griesheim, Germany). Animals were sacrificed by cervical dislocation upon reaching the humane endpoint (tumor volume ≥ 1.500 mm^3^; body weight loss ≥ 15%). Statistical analysis was performed using GraphPad Prism software, applying one-way ANOVA followed by Tukey’s test. To evaluate the cooperativity of combination treatment the expected additivity was calculated according to the Bliss additivity model (C = A + B – A x B; wherein C is the expected T/C of the combination of drug A and drug B if they act additive, A is T/C of drug A, and B is T/C of drug B). Ten percent over the expected additive effect is assumed to indicate synergism of the two drugs and 10% below the expected additive effect is assumed to indicate antagonism [36].

Immunohistochemistry: HER2 expression was determined by immunohistochemical (IHC) staining using antibody EP1045Y rabbit monoclonal antibody, (Abcam, Cambridge, UK), followed by incubation with HRP-anti-rabbit labeled polymer (Envision, Dako/Agilent, Santa Clara, USA)) and subsequent visualization using using 3,3′-diaminobenzidine. In parallel, xenograft samples were stained with an isotype control antibody to demonstrate specificity of the staining (rabbit normal serum, Dako/Agilent, Santa Clara USA).

## 5. Patents

The work is included in a submitted patent.

## Figures and Tables

**Table 1:** Summary of in vitro cellular experiments, biodistribution, and in vivo efficacy studies, using the isogenic cell line pair DLD-1 parental and DLD-1 BRCA2 -/-. In vitro experiments comprised determination of cell surface HER2 antigens by flow cytometry and cytotoxicity experiments. In vivo, the biodistribution of HER2-TTC was compared to radiolabeled isotype control. Respective tumors to blood ratios 336 h after start of treatment are presented. Treatment over control ratio, including statistical significance compared to vehicle, were determined from the respective efficacy studies.

	Tissue Type	DLD-1 Parental	DLD-1 BRCA2 -/-
		Colorectal Adenocarcinoma	
Cancer model characteristics	mAb/receptor	6500	3400
	Average doubling time in vitro (h)/in vivo (d)	35/7.3	57/8.3
	HER2 IHC score	1—2+	1—2+
In vitro cytotoxicity	IC_50_ (kBq/mL HER2-TTC)	22	3
	IC_50_ (pM ^227^Th)	86	10
	IC_50_ (µM olaparib)	5	0.03
	Average combination index (CI)	1	0.6
Biodistribution	Tumor/blood ratio (HER2-TTC, 336 h)	12.8 ± 2.4	17.3 ± 3.5
	Tumor/blood ratio (isotype control, 336 h)	1.2	1.5
Treatment/Control In vivo Efficacy	HER2-TTC, 125 kBq/kg bw	0.9 (n.s.)	0.8 (n.s.)
	HER2-TTC, 300 kBq/kg bw	0.5 (*p* = 0.03)	0.5 (*p* < 0.0001)
	HER2-TTC, 600 kBq/kg bw	0.3 (*p* < 0.0001)	0.1 (*p* < 0.0001)
	Olaparib 25 mg/kg bw	0.6 (n.s.)	0.4 (*p* < 0.0001)
	Olaparib 50 mg/kg bw	0.6 (n.s.)	0.5 (*p* < 0.0001)
	125 kBq/kg bw + 25 mg/kg bw olaparib	0.8 (n.s.)	0.4 (*p* < 0.0001)
	125 kBq/kg bw + 50 mg/kg bw olaparib	0.6 (n.s.)	0.1 (*p* < 0.0001)
	300 kBq/kg bw + 25 mg/kg bw olaparib	0.4 (*p* < 0.0001)	0.2 (*p* < 0.0001)
	300 kBq/kg bw + 50 mg/kg bw olaparib	0.5 (*p* = 0.009)	0.03 (*p* < 0.0001)

## Supplementary Materials

The following are available online at https://www.mdpi.com/1424-8247/12/4/155/s1, Figure S1: Analysis of HER2-TTC by SEC-HPLC upon storage. Figure S2: Tumor uptake and retention in DLD-1 BRCA2 -/- compared to DLD-1 parental. Figure S3. Measured activity of thorium-227 and radium-223 in Bq/g in tumors isolated from biodistribution studies. Figure S4: Body weights of DLD-1 parental or DLD-1 BRCA2-/- bearing mice. Table S1. Radiostability of HER2-TTC over the course of 48 h at room temperature. Table S2. Overview of determined % IA/g from biodistribution studies in the DLD-1 parental and DLD-1 BRCA2 -/- xenograft models. Table S3: BLISS analysis DLD-1 BRCA2 -/-. Table S4: BLISS analysis DLD-1 parental.
