# Supplementary material for: Synergistic Effect of a HER2 Targeted Thorium-227 Conjugate in Combination with Olaparib in a BRCA2 Deficient Xenograft Model
Source: Pharmaceuticals (Basel). 2019 Oct 15;12(4):155. doi: 10.3390/ph12040155 (PMC6958469; doi:10.3390/ph12040155)
Supplement: Supplementary file 1 [file pharmaceuticals-12-00155-s001.pdf]

Supporting Information for:

"Synergistic Effect of a HER2 Targeted Thorium-227 Conjugate (TTC) in combination with olaparib in a BRCA2 -/- deficient xenograft model"

Katrine Wickstroem [1], Jenny Karlsson [1*], Christine Ellingsen [1], Véronique Cruciani [1], Alexander Kristian [1], Urs B Hagemann [2], Roger M Bjerke [1], Olav B Ryan [1], Lars Linden [3], Dominik Mumberg [2], Michael Brands [2] and Alan Cuthbertson [1]

[1] Thorium Conjugate Research, Bayer AS, Oslo, Norway

[2] Bayer AG, Pharmaceuticals Division, Berlin, Germany

[3] Bayer AG, Pharmaceuticals Division, Wuppertal, Germany

* Correspondence: jenny.karlsson@bayer.com; Tel.: +47 4134 0712 (J.K.)

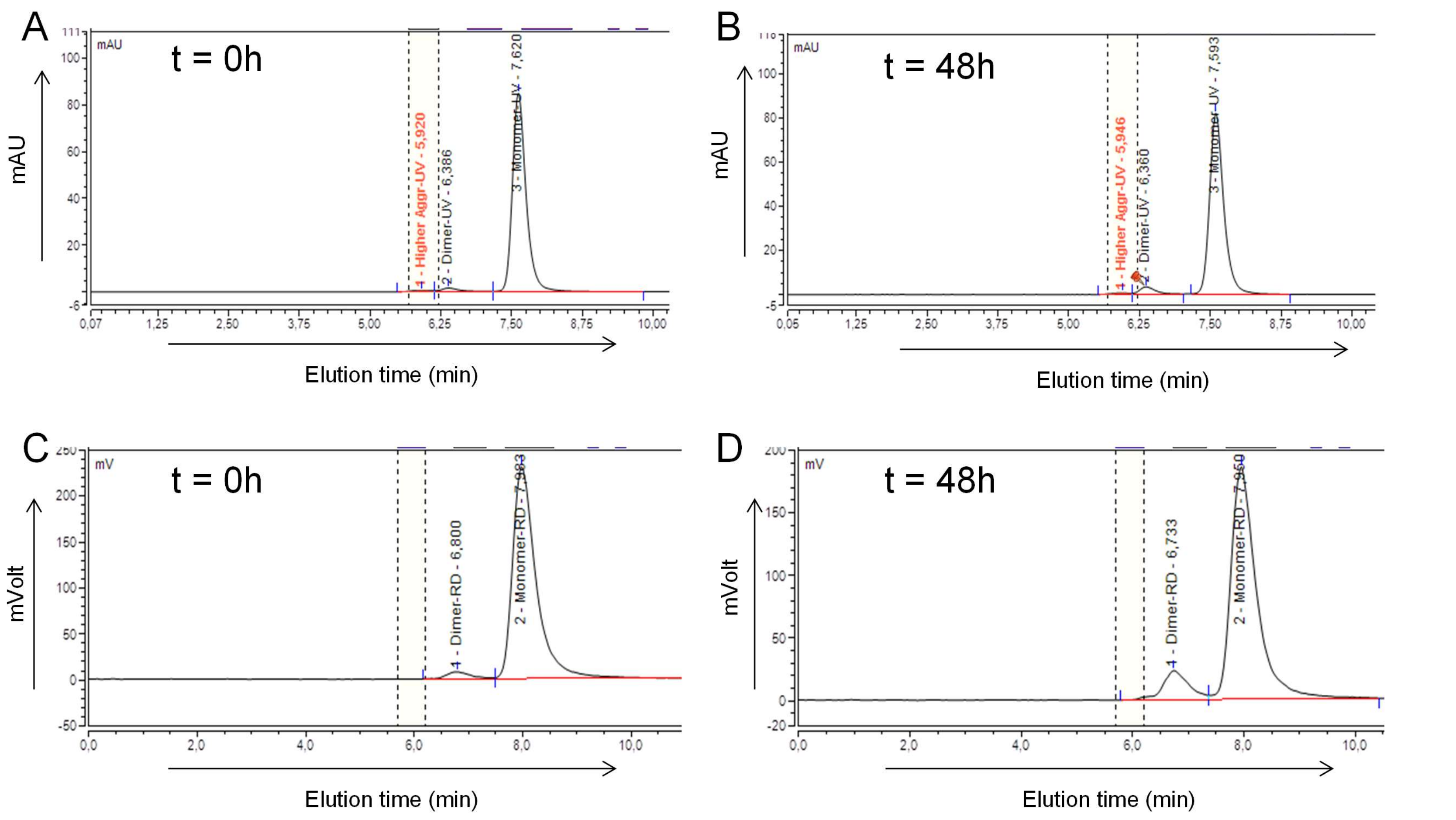

Figure S1. Analysis of HER2-TTC by SEC-HPLC upon storage. HER2-TTC was radiolabeled at a specific activity of 2 kBq/µg at an radioactive concentration of 2 MBq/ml and stored at room temperature. At t = 0 and 48 h, aliquots were analyzed by SEC-HPLC. Absorbance at UV 280 nm served to monitor changes in monomer levels **(A, B)**. Similarly, radio-HPLC **(C, D)** served to monitor changes in monomer levels in the radiolabeled fraction of HER2-TTC.

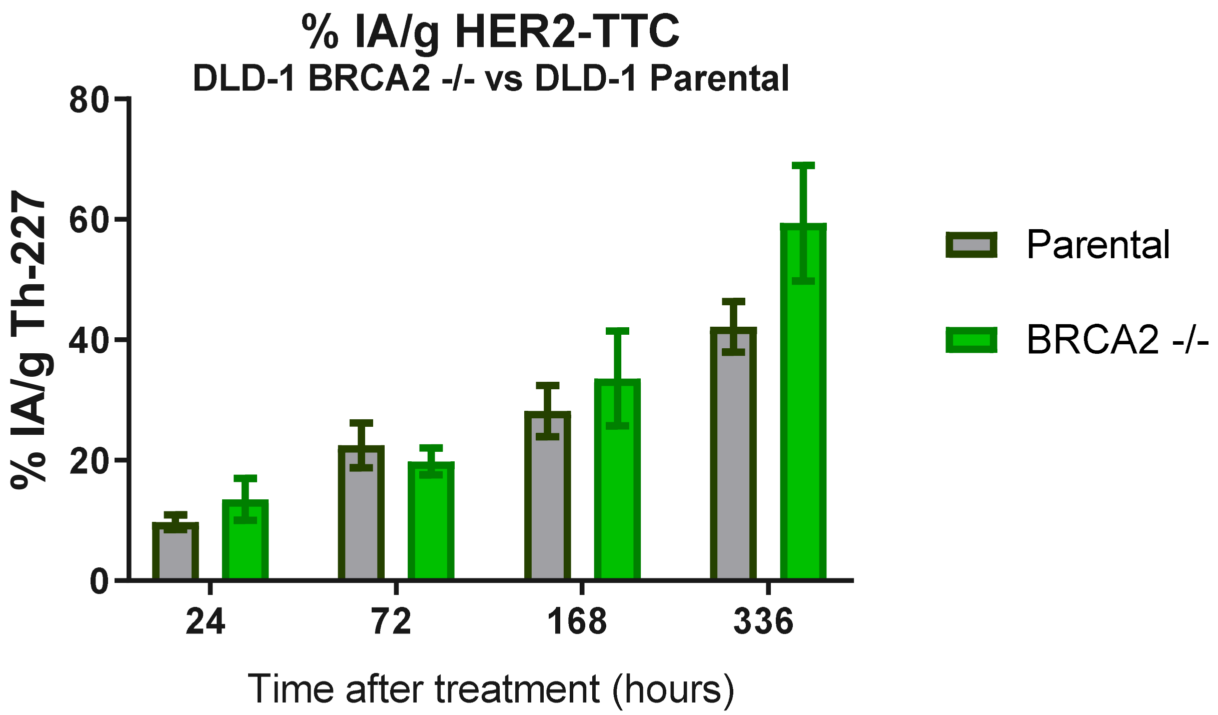

Figure S2. Tumor uptake and retention in DLD-1 BRCA2 -/- compared to DLD-1 Parental. Percentage of injected activity of thorium-227 per gram (% IA/g) in DLD-1 parental and DLD-1 BRCA2 -/- tumors after a single treatment with HER2-TTC (600 kBq/kg). The % IA/g was not significantly different between the models at any of the the timepoints.

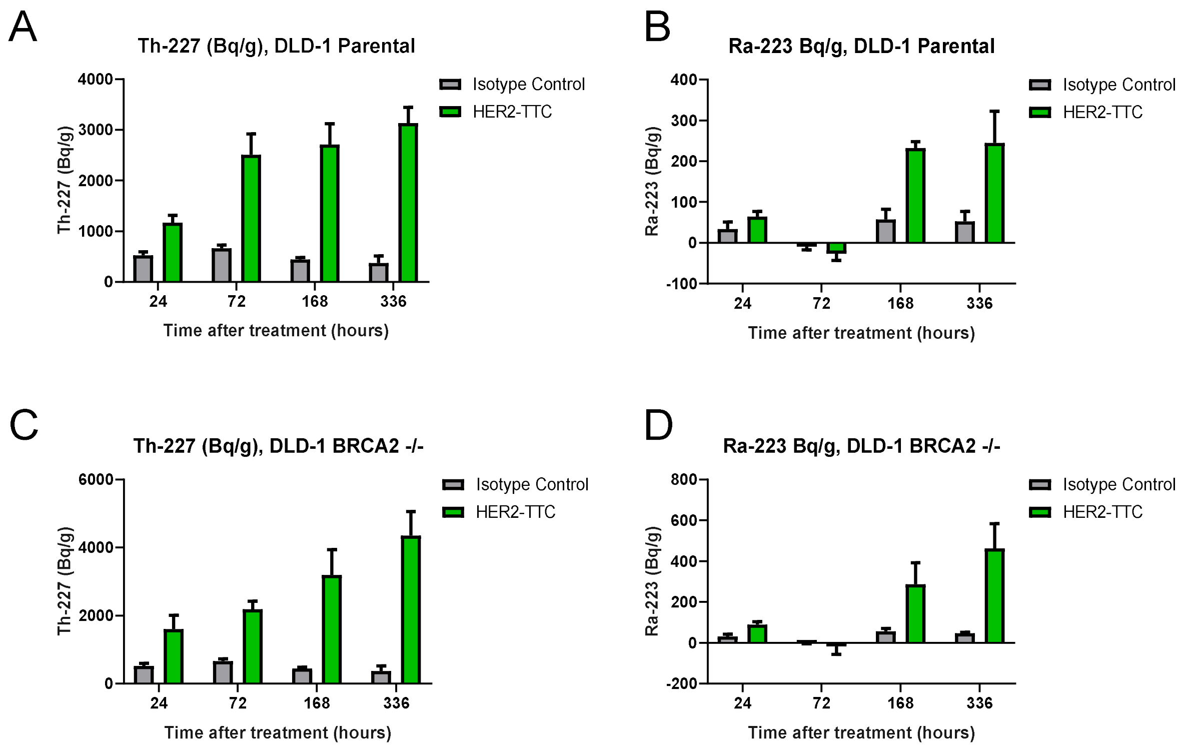

Figure S3. Measured activity of thorium-227 and radium-223 in Bq/g in tumors isolated from biodistribution studies. **A** and **B)** Th-227 or Ra-223 activity in DLD-1 parental tumors. **C** and **D)** Th-227 or Ra-223 activity in DLD-1 BRCA2 -/- tumors.

A

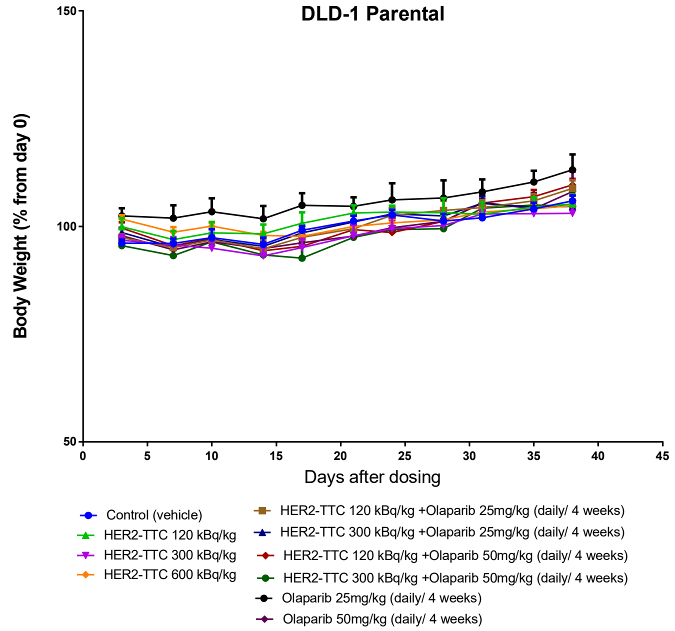

B

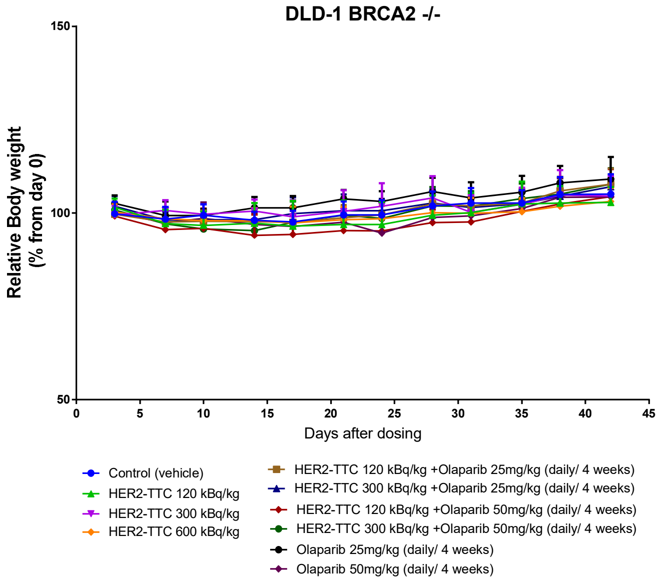

Figure S4. Body weights of DLD-1 parental or DLD-1 BRCA2-/- bearing mice. Body weight in mice with **A)** DLD-1 parental xenograft and **B)** DLD-1 BRCA2 -/- xenograft bearing mice after a single dose administration of HER2-TTC (120, 300 or 600 kBq/kg, 0.14 mg/kg, i.v.) and olaparib (25 or 50 mg/kg daily for four weeks) as monotherapies or in combination treatment as compared to vehicle.

Table S1. Radiostability of HER2-TTC over the course of 48 hours at room temperature. HER2-TTC was radiolabeled at a specific activity of 2 kBq/μg at and a radioactive concentration of 2 MBq/ml.

	**Radiochemical Purity**		**Binding**
**Time (h)**	**ITLC (%)**	**HPLC (%)**	**IRF***
0.5	94	97	n.d.
1	98	96	n.d.
2	98	98	74
6	99	97	n.d.
24	99	96	72
48	99	95	77

*, Immuno-Reactive Fraction

Table S2. Overview of determined % IA/g from biodistribution studies in the DLD-1 parental and DLD-1 BRCA2 -/- xenograft models.

DLD-1 parental								
HER2-TTC	24h		72h		168h		336h	
% IA/g*	*Average*	*SD*	*Average*	*SD*	*Average*	*SD*	*Average*	*SD*
Blood	17,1	1,6	11,3	0,4	6,3	0,4	3,4	1,1
Tumor	9,7	1,2	22,5	3,7	28,2	4,3	42,2	4,2
Femur	4,8	0,4	6,6	0,8	8,4	0,6	10,0	0,5
Kidney	5,5	0,3	7,3	2,6	6,4	0,4	5,0	0,4
Liver	5,4	0,0	5,9	0,8	6,2	1,1	6,6	0,3
Spleen	4,7	0,2	7,7	1,1	7,4	3,4	6,5	3,2

Isotype Control	24h		72h		168h		336h	
% IA/g	*Average*	*SD*	*Average*	*SD*	*Average*	*SD*	*Average*	*SD*
Blood	18,5	2,0	15,3	1,1	7,1	2,3	4,2	2,6
Tumor	4,4	0,6	6,1	0,6	4,7	0,4	5,1	2,0
Femur	3,6	0,5	7,2	0,9	6,7	1,0	10,7	2,2
Kidney	4,6	0,3	7,8	0,7	5,2	1,3	4,4	2,0
Liver	4,8	0,4	5,4	0,4	5,8	1,5	5,7	1,5
Spleen	5,0	0,3	10,2	1,7	9,4	1,6	5,8	2,4

DLD-1 BRCA2 -/-								
HER2-TTC	24h		72h		168h		336h	
% IA/g	*Average*	*SD*	*Average*	*SD*	*Average*	*SD*	*Average*	*SD*
Blood	15,8	0,6	10,0	1,9	6,0	1,8	3,5	0,7
Tumor	13,5	3,5	19,8	2,2	33,6	7,9	59,4	9,6
Femur	4,8	0,4	5,4	0,9	6,8	1,3	9,7	0,3
Kidney	4,1	0,1	5,2	1,5	5,1	0,9	5,0	1,0
Liver	4,6	0,2	4,3	0,7	5,0	1,1	5,4	1,0
Spleen	4,5	0,9	6,4	1,3	8,9	4,0	10,8	1,8

Isotype Control	24h		72h		168h		336h	
% IA/g	*Average*	*SD*	*Average*	*SD*	*Average*	*SD*	*Average*	*SD*
Blood	16,7	1,1	15,5	1,2	8,6	0,5	4,1	1,1
Tumor	5,0	0,2	6,2	0,3	6,4	0,5	5,9	0,4
Femur	3,7	0,3	6,8	0,8	7,6	0,8	9,7	0,7
Kidney	4,3	0,4	6,6	0,4	6,3	0,9	5,2	1,0
Liver	4,9	0,5	6,5	0,6	6,1	0,7	7,6	1,7
Spleen	4,9	0,9	11,9	0,7	12,8	2,5	8,6	0,4

*****, injected activity of thorium-227 per gram

Table S3. BLISS analysis DLD-1 BRCA2 -/-.

	T/C [%]	Inhibition [%]	Calculated combined response [%]	Excess over Bliss additivity [%]
**Olaparib (25 mg/kg)**	40.	60	0.6	NA
**HER2-TTC (120 kBq/kg)**	80	20	0.2	NA
**HER2-TTC (120 kBq/kg) + olaparib (25 mg/kg)**	40	60	0.7	-12

	T/C [%]	Inhibition [%]	Calculated combined response [%]	Excess over Bliss additivity [%]
**Olaparib (25 mg/kg)**	40	60	0.6	NA
**HER2-TTC (300 kBq/kg)**	50	50	0.5	NA
**HER2-TTC (300 kBq/kg) + olaparib (25 mg/kg)**	20	80	0.8	0

	T/C [%]	Inhibition [%]	Calculated combined response [%]	Excess over Bliss additivity [%]
**Olaparib (50 mg/kg)**	50	50	0.5	NA
**HER2-TTC (120 kBq/kg)**	80	20	0.2	NA
**HER2-TTC (120 kBq/kg) + olaparib (50 mg/kg)**	10	90	0.6	50

	T/C [%]	Inhibition [%]	Calculated combined response [%]	Excess over Bliss additivity [%]
**Olaparib (50 mg/kg)**	50	50	0.5	NA
**HER2-TTC (300 kBq/kg)**	50	50	0.5	NA
**HER2-TTC(300 kBq/kg)+ olaparib (50 mg/kg)**	3	97	0.8	29

Table S4. BLISS analysis DLD-1 Parental.

	T/C [%]	Inhibition [%]	Calculated combined response [%]	Excess over Bliss additivity [%]
**Olaparib (25 mg/kg)**	60	40	0.4	NA
**HER2-TTC (120 kBq/kg)**	90	10	0.1	NA
**HER2-TTC (120 kBq/kg) + olaparib (25 mg/kg)**	80	20	0.5	-57

	T/C [%]	Inhibition [%]	Calculated combined response [%]	Excess over Bliss additivity [%]
**Olaparib (25 mg/kg)**	60	40	0.4	NA
**HER2-TTC (300 kBq/kg)**	50	50	05	NA
**HER2-TTC (300 kBq/kg) + olaparib (25 mg/kg)**	40	60	0.7	-14

	T/C [%]	Inhibition [%]	Calculated combined response [%]	Excess over Bliss additivity [%]
**Olaparib (50 mg/kg)**	60	40	0.4	NA
**HER2-TTC (120 kBq/kg)**	90	10	0.1	NA
**HER2-TTC (120 kBq/kg) + olaparib (50 mg/kg)**	60	40	0.5	-13

	T/C [%]	Inhibition [%]	Calculated combined response [%]	Excess over Bliss additivity [%]
**Olaparib (50 mg/kg)**	60	40	0.4	NA
**HER2-TTC (300 kBq/kg)**	50	50	0.5	NA
**HER2-TTC(300 kBq/kg)+ olaparib (50 mg/kg)**	50	50	0.7	-29
